# Bench and mathematical modeling of the effects of breathing a helium/oxygen mixture on expiratory time constants in the presence of heterogeneous airway obstructions

**DOI:** 10.1186/1475-925X-11-27

**Published:** 2012-05-30

**Authors:** Andrew R Martin, Ira M Katz, Karine Terzibachi, Laure Gouinaud, Georges Caillibotte, Joëlle Texereau

**Affiliations:** 1 Senior Research Scientist, Medical Gases, American Air Liquide, Delaware Research and Technology Center (DRTC), 200 GBC Drive, Newark, DE, 19702, USA; 2 Medical Gases Group, Air Liquide Santé International, Les Loges-en-Josas, 78354, France; 3 Department of Mechanical Engineering, Lafayette College, Easton, PA, 18042, USA

**Keywords:** Helium, Heliox, Exhalation, Time constant, Hyperinflation, Gas trapping, Ventilation distribution, Airway resistance, Mechanical ventilation, Lung

## Abstract

**Background:**

Expiratory time constants are used to quantify emptying of the lung as a whole, and emptying of individual lung compartments. Breathing low-density helium/oxygen mixtures may modify regional time constants so as to redistribute ventilation, potentially reducing gas trapping and hyperinflation for patients with obstructive lung disease. In the present work, bench and mathematical models of the lung were used to study the influence of heterogeneous patterns of obstruction on compartmental and whole-lung time constants.

**Methods:**

A two-compartment mechanical test lung was used with the resistance in one compartment held constant, and a series of increasing resistances placed in the opposite compartment. Measurements were made over a range of lung compliances during ventilation with air or with a 78/22% mixture of helium/oxygen. The resistance imposed by the breathing circuit was assessed for both gases. Experimental results were compared with predictions of a mathematical model applied to the test lung and breathing circuit. In addition, compartmental and whole-lung time constants were compared with those reported by the ventilator.

**Results:**

Time constants were greater for larger minute ventilation, and were reduced by substituting helium/oxygen in place of air. Notably, where time constants were long due to high lung compliance (i.e. low elasticity), helium/oxygen improved expiratory flow even for a low level of resistance representative of healthy, adult airways. In such circumstances, the resistance imposed by the external breathing circuit was significant. Mathematical predictions were in agreement with experimental results. Time constants reported by the ventilator were well-correlated with those determined for the whole-lung and for the low-resistance compartment, but poorly correlated with time constants determined for the high-resistance compartment.

**Conclusions:**

It was concluded that breathing a low-density gas mixture, such as helium/oxygen, can improve expiratory flow from an obstructed lung compartment, but that such improvements will not necessarily affect time constants measured by the ventilator. Further research is required to determine if alternative measurements made at the ventilator level are predictive of regional changes in ventilation. It is anticipated that such efforts will be aided by continued development of mathematical models to include pertinent physiological and pathophysiological phenomena that are difficult to reproduce in mechanical test systems.

## Background

Resistance to expiratory flow is a concern for patients with obstructive lung disease. Thickening of airway walls, partial occlusion of lumen due to excessive mucus production, and airway narrowing associated with smooth muscle contraction can all contribute to increased airway resistance, which impedes expiratory flow and can lead to dynamic hyperinflation and dyspnea [1-4]. Previous studies have explored the hypothesis that breathing low-density helium/oxygen (He/O_2_) mixtures improves lung emptying in patients with obstructive lung disease [5-9]. From these investigations, coupled with analysis of the underlying respiratory fluid mechanics, it may be gathered that the potential for He/O_2_ to influence expiratory flow rates is highly dependent on the location of obstruction [5,6]. Moving from the upper airways to the peripheral lung, as the gas Reynolds number decreases by several orders of magnitude, airway resistance becomes less influenced by flow inertia (which depends in turn on gas density) and more influenced by viscous effects [5,10,11].

In addition to their depth along the respiratory tract, airway obstructions may also vary in their severity across different airways at the same depth in the lung [12,13]. Variation in airway resistance between lung regions leads to regional differences in ventilation, including differences in expiratory time constants. In such circumstances, end-expiratory volumes and pressures will also vary regionally [5,14]. As noted recently by Diehl and colleagues [15], breathing He/O_2_ may hypothetically modify regional time constants so as to redistribute ventilation. Previously, we employed a dual chamber mechanical test lung to demonstrate that, in the presence of heterogeneous airway resistance representative of conducting airway obstruction, the ventilation distribution between chambers on inspiration was more homogeneous for He/O_2_ than for air [11]. In the present work, we used a similar approach to investigate the influence of He/O_2_ on expiratory flow, with attention paid to differences between the behavior of an individual, obstructed lung compartment and the lung as a whole. Additional measurements were made to assess the expiratory resistance imposed by the breathing circuit of the ventilator used to supply gas to the test lung, and these data were used as input in the development of an analytical model describing the test system.

## Methods

### Bench experiments

**Figure 1:**
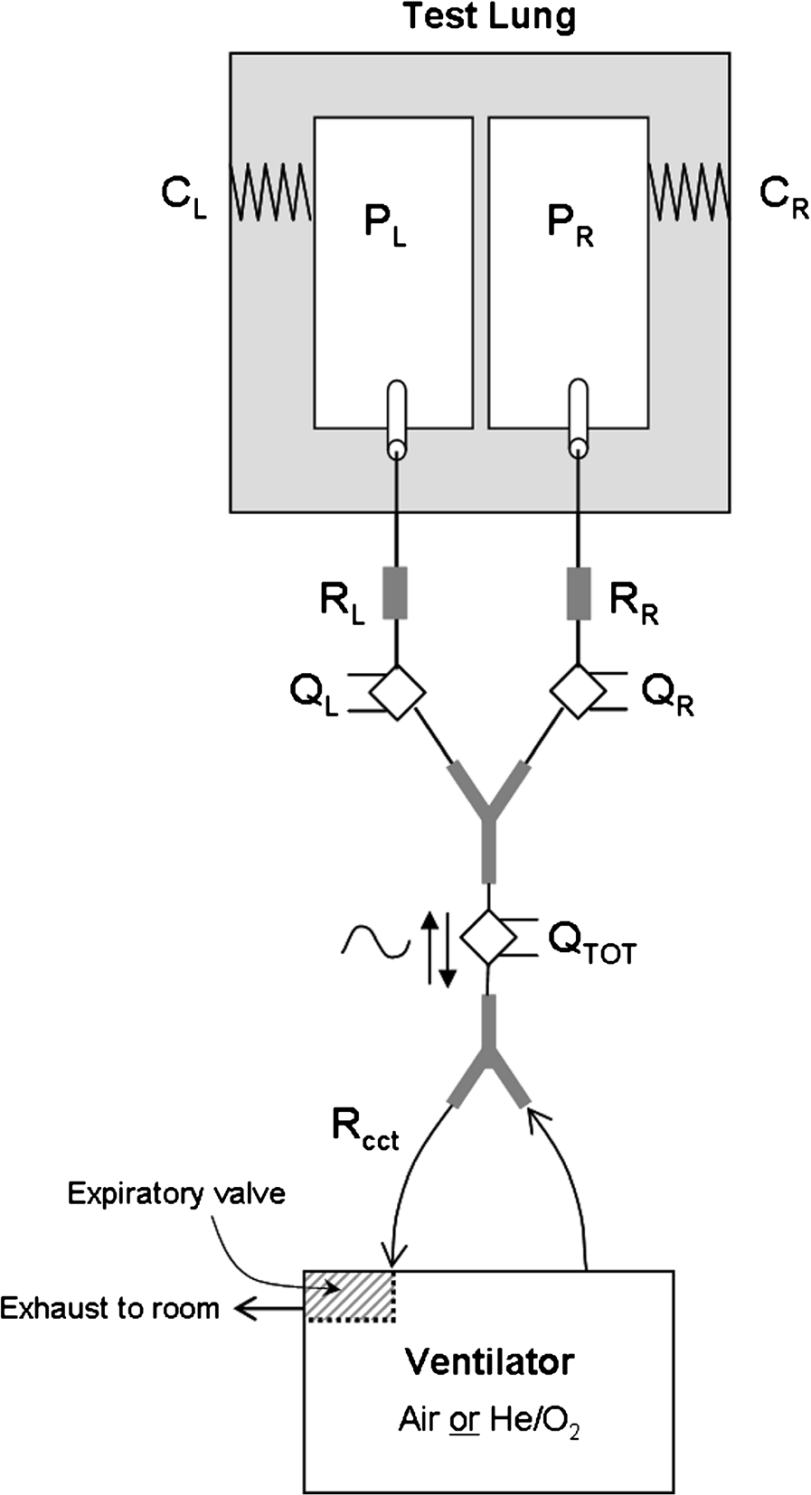
**Schematic diagram of the experimental apparatus.** The subscripts L and R refer to the left and right branches of the test lung. C refers to the compliance of either compartment of the test lung, P is the compartmental pressure, R is the resistance inserted into either branch of the test lung, or that imposed by the external breathing circuit (R_cct_), and Q is the flow in either branch, or the total flow into and out of the test lung (Q_TOT_).

The bench apparatus was similar to that described previously by Katz et al. [11]. As displayed schematically in Figure 1, the two compartments of a dual adult test lung (Michigan Instruments, USA) were connected through a symmetric Y-piece to the patient end of a ventilator breathing circuit. Flows into and out of the compartments (Q_L_ and Q_R_) were measured using variable orifice flow sensors (PN 155362; Hamilton Medical AG, Switzerland) identical to that used by the ventilator itself to monitor the total flow (Q_TOT_). Differential pressure signals across the two compartmental flow sensors were obtained using low pressure transducers (PX277-05D5V; Omega Engineering Inc., USA) and processed using LabVIEW software (National Instruments Corp., USA) to produce flow versus time curves. Prior to the experiments, the left and right flow sensors were calibrated against known flow rates of medical air and He/O_2_ provided by a mass flow controller (EL-FLOW Select; Bronkhorst High-Tech, Netherlands). The ventilator flow sensor was calibrated with medical air or He/O_2_ prior to each experiment following the ventilator’s standard calibration procedure.

The test lung was ventilated in volume-control mode with either dry medical air (78% N_2_/22% O_2_; Air Liquide, France) or He/O_2_ (78% He/22% O_2_; Air Liquide, France) using a Hamilton G5 ventilator (Hamilton Medical AG, Switzerland). Two breathing patterns were used: a 500 ml tidal volume at 12 breaths/min, producing a minute ventilation (V_E_) of 6 l/min, and a 1000 ml tidal volume at 20 breaths/min, producing a minute ventilation of 20 l/min. In either case, the inspiratory/expiratory ratio was ½, and flow was set as constant over the inspiratory phase (i.e., a square wave).

Parabolic resistors (PneuFlow; Michigan Instruments, USA) were placed in the two limbs of the test lung (R_L_ and R_R_). While R_L_ was held constant with an Rp5 resistor, R_R_ was varied between an Rp5, Rp20, or Rp50. These resistors obey the relationship

(1) $$\Delta P=\frac{k}{2}\rho {\bar{U}}^{2}$$

where *ΔP* is the pressure loss across the resistor, *ρ* is the gas density, ${\bar{U}}^{}$is the gas velocity averaged over the cross-section of the entrance to the resistor, and k is a loss coefficient that, over the conditions tested, is a function of only the geometry of the resistor. Table 1 displays values of k for the parabolic resistors, along with equivalent values of the linear resistance R for air and He/O_2_ at two representative flow rates. The compliances of the left and right compartments (C_L_ and C_R_) were at all times equal to one another, but were varied between 0.02, 0.05, and 0.10 l/cm H_2_O over different experiments.

**Table 1:** Loss Coefficients (k) and Equivalent Linear Resistances (R) for the Rp5, Rp20, and Rp50 Parabolic Resistors

			**R[cmH**_**2**_**OI**^**-1**^** s]**	
**Resistor**	**k**	**Q[I/min]**	**Air**	**He/O**_**2**_
Rp5	3.3	20	0.9	0.3
		60	2.7	0.9
Rp20	21.5	20	5.8	2.0
		60	17.5	6.1
Rp50	132.9	20	36.1	12.7
		60	108.4	38.0

Finally, in order to provide input data for the analytical model, additional measurements were performed to assess the resistance imposed by the flow sensors and the expiratory limb of the breathing circuit. The pressure drop across either a single flow sensor, from inlet to outlet, or the entire expiratory circuit, from the patient end of the ventilator’s proximal flow sensor through the expiratory valve, was measured using a digital manometer (PR-201; Eurolec, Ireland) over a range of steady flow rates of medical air or He/O_2_ supplied by the mass flow controller.

### Calculation of time constants

The single-compartment linear model of the respiratory system has been widely employed as a conceptual framework through which to discuss and explain various observed phenomena related to breathing mechanics. This model reduces the respiratory system to a single compliant compartment supplied by a single airway of a specified resistance. For the single-compartment linear model, the driving pressure across the system is given by Otis et al. [16]:

(2) $$\Delta P=\frac{1}{C}V+RQ$$

where Δ*P* is the driving pressure, *V* is the volume of the compartment, *Q* is the volumetric flow rate into or out of the system, and *R* and *C* are constants representing, respectively, the resistance and compliance of the system. The resistance is specified as the pressure drop across the airway per unit flow rate, whereas the compliance is the change in compartment volume per unit pressure.

For a single-compartment linear model of the respiratory system, the time-dependence of the expiratory flow rate can be described as purely monoexponential [17]:

(3) $$Q_{e}\left(t\right)=Q_{e,p}e^{\frac{-t}{\tau }}$$

where *Q*_*e*_(*t*) is the expiratory flow rate at time *t*, *Q*_*e,p*_ is the peak expiratory flow rate, and the time constant:

(4) τ=RC

is the product of the resistance and compliance of the system. This time constant also describes the time-dependence of the volume response of such as system:

(5) $$V_{e}\left(t\right)=\tau Q_{e,p}\left(1-e^{\frac{-t}{\tau }}\right)$$

where *V*_*e*_(*t*) is the exhaled volume at time t.

In the present work, a two-compartment model with non-linear resistance was employed, for which expiratory flow did not follow the idealized exponential form of Equation 3. Nevertheless, *apparent*, or representative, time constants were calculated for each compartment, and for the test lung as a whole, following the iterative method described by Brunner et al. [18]. Equation (5) can be evaluated at the end of expiration and rearranged to yield:

(6) $$\tau =\frac{V_{e,tot}}{Q_{e,p}}{\left(1-e^{\frac{-t_{e}}{\tau }}\right)}^{-1}$$

where *V*_*e,tot*_ is the total exhaled volume, and *t*_*e*_ is the exhalation time.

Equation (6) is an implicit equation for the time constant, which was solved iteratively with an initial estimate of:

(7) $$\tau_{0}=\frac{V_{e,tot}}{Q_{e,p}}$$

followed by iterations of the form:

(8) $$\tau^{k+1}=\tau_{0}{\left(1-e^{-t_{e}/\tau^{k}}\right)}^{-1}$$

The solution described in Equations (7) and (8) was implemented in Microsoft Excel, with input values of *V*_*e,tot*_, *Q*_*e,p*_, and *t*_*e*_ obtained from flow versus time data acquired for each compartment, and the whole lung, as described above. It was found that the time constant calculated in this manner typically varied between iterations by less than 1% after no more than 3 or 4 iterations.

Under the controlled experimental conditions described above, there was only very small variation in the calculated time constant between repetitions performed with all parameters held constant; therefore, for the experimental time constants reported below, uncertainty in measurement was estimated as the maximum difference between values determined for the right and left compartment when an Rp5 was inserted in both limbs (in which cases the time constants for the left and right compartments should ideally have been identical, and differences were attributed mainly to experimental uncertainties in the set left and right chamber compliances).

### Analytical model

In contrast to the idealized single-compartment linear model, a closed form solution of exhalation for the two-compartment lung model with nonlinear resistance is not possible. Therefore, the problem was solved numerically using MATLAB (Mathworks, United States). The analysis followed the engineering pressure loss model presented previously by Katz et al. [11], where in the present case the energy balance was made between each chamber and the atmosphere after accounting for losses through the complete expiratory path including the parabolic resistors, flow meters, Y-piece, breathing circuit, and the ventilator’s expiratory valve (Figure 1). Accordingly, with the atmospheric pressure equal to zero, the governing equation for either the left or right chamber was:

(9) $$\frac{P_{i}}{\rho }=\frac{V_{i}}{\rho C_{i}}=\frac{\left(k_{i}+k_{\mathrm{fs}}\right)}{2}{\left(\frac{Q_{i}}{A}\right)}^{2}+\frac{k_{\mathrm{cct}}}{2}{\left(\frac{Q_{i}+Q_{j}}{A}\right)}^{2}$$

where the subscript *i* indicates the left or right chamber, the subscript *j* indicates the opposite chamber, *P* is the chamber pressure, *V* is the volume of gas in the chamber, *C* is the chamber compliance, *Q* is the volumetric flow rate out of the chamber, *A* is the cross-sectional area of the airway, and *k*_*i*_, *k*_*fs*_, and *k*_*cct*_ represent loss coefficients for the parabolic resistor, the flow sensor, and the expiratory circuit, respectively. Of note, unlike the loss coefficients for the parabolic resistors provided in Table 1, loss coefficients for the flow sensors and expiratory limb of the breathing circuit depended on gas density and flow rate. Therefore, the measurements described above in Section 2.1 for the resistances imposed by these components were fit with second order polynomials for use in the analytical model.

Equation (9) was solved for either chamber with the initial chamber volume and pressure at the start of exhalation calculated using the inhalation model of Katz et al. [11]. The analysis consisted of a quasi-steady solution for the flow rates at each time step, progressing until the expiratory time limit was reached. Time constants for the left and right chambers were again determined according to equations (7) and (8), in this case using the peak flow and exhaled volume determined by the model.

## Results and discussion

### Time constants determined experimentally

**Figure 2:**
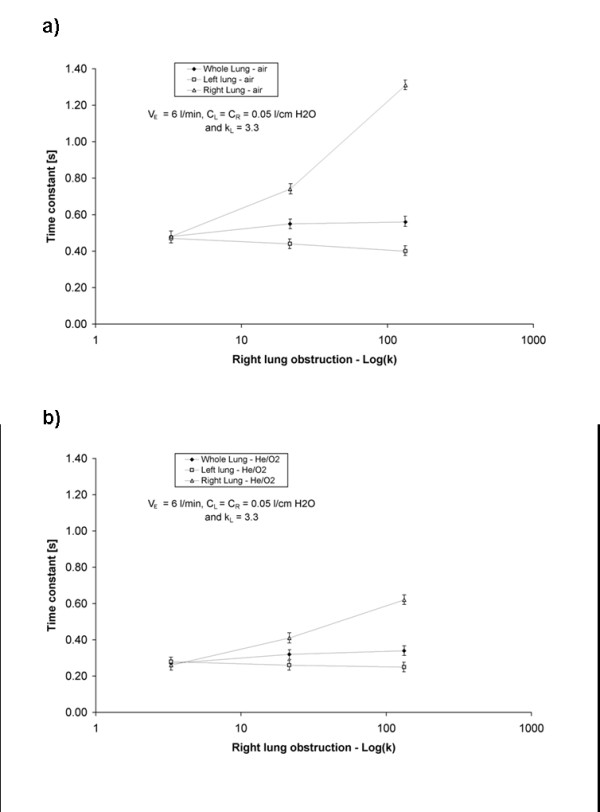
**Experimentally determined expiratory time constants for the right, left, and whole lung plotted against the log of the loss coefficient of the right lung obstruction.** The left lung obstruction was held constant at k_L_ = 3.3. The compliances of the left and right chambers were both equal to 0.05 l/cm H_2_O. The test lung was ventilated with a tidal volume of 500 ml at 12 breaths/min with (**a**) air or (**b**) He/O_2_ to produce a minute ventilation of 6 l/min.

**Figure 3:**
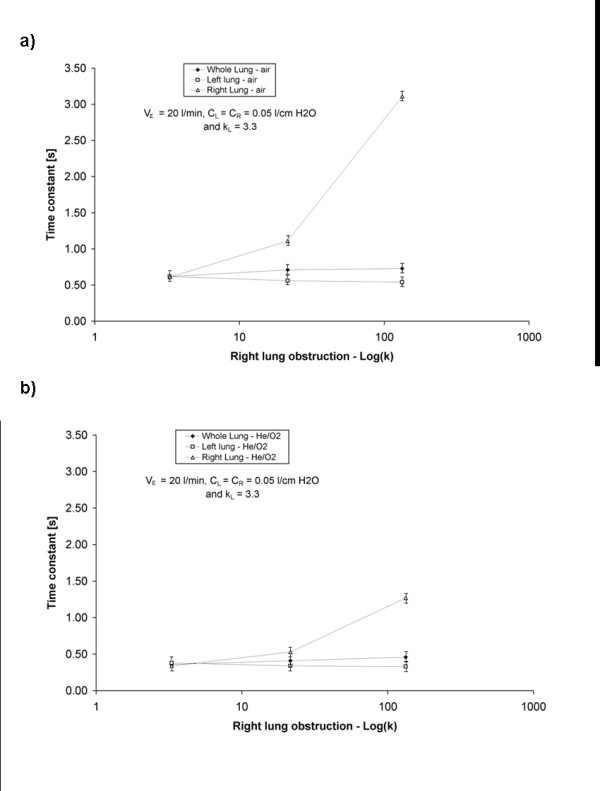
**Experimentally determined expiratory time constants for the right, left, and whole lung plotted against the log of the loss coefficient of the right lung obstruction.** The left lung obstruction was held constant at k_L_ = 3.3. The compliances of the left and right chambers were both equal to 0.05 l/cm H_2_O. The test lung was ventilated with a tidal volume of 1000 ml at 20 breaths/min with (**a**) air or (**b**) He/O_2_ to produce a minute ventilation of 20 l/min.

For each set of experimental conditions, expiratory time constants were determined for the left and right chambers of the test lung from measured expiratory flow versus time data. In addition, a whole-lung time constant was determined from the total expiratory flow out of the test lung. Figures 2 and 3 display, for the two breathing patterns studied, changes in compartmental and whole lung time constants as the right chamber was increasingly obstructed. For a given breathing pattern and gas, the time constant of the right chamber increased as the severity of obstruction in the right branch of the test lung was increased. However, the increase in the whole lung time constant was much less pronounced, and coincided with a slight decrease in the time constant of the left chamber. The latter effect was unexpected, but we speculate that it resulted from the pendelluft phenomenon described by Otis et al. [16]. As the severity of obstruction in the right lung increased, cycling between inspiration and expiration in the right and left compartments was observed to become increasingly out of phase, such that the relatively unobstructed left lung began to expire gas while the right lung was still inspiring. This presented a flow path (from the left to the right compartment) for a small portion of gas expired from the left lung that had lower resistance than the normal flow path through the ventilator’s expiratory circuitry, and therefore may explain the influence of right chamber obstruction on left chamber time constants observed in Figures 2 and 3.

Comparing Figure 2 and Figure 3 further, it is clear that, all else being equal, measured time constants were greater at higher minute ventilation, and lower for He/O_2_ than for air. The former effect is no doubt due to the flow-dependence of resistance exhibited by the parabolic resistors used in the present study, as evidenced above in Table 1. Regarding the effect of He/O_2_, the pressure drop across these resistors results from phenomena that are inertial in nature, and therefore density-dependant [11]. By substituting He/O_2_ in place of air, the low density of the mixture reduces resistance, in turn reducing expiratory time constants. The applicability of these results to the human respiratory tract, where airway resistance contains both inertial and viscous components, will clearly depend on the nature and severity of airway disease, with obstructions occurring in more proximal airways favoring inertial effects, and those in the distal airways favoring viscous effects. However, as discussed below, it should be noted that the resistances imposed by healthy, conducting airways, and by common components of external breathing circuits, are both flow- and density-dependent.

**Figure 4:**
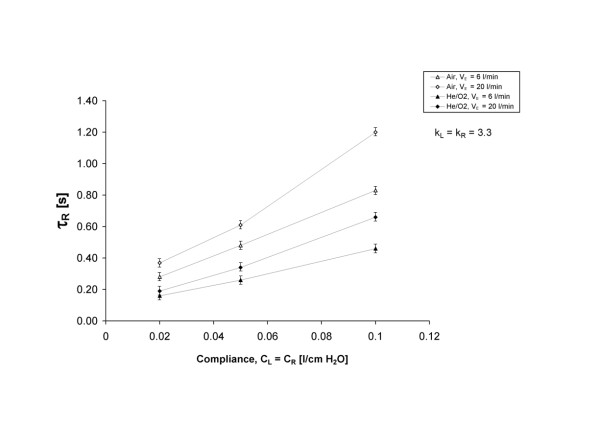
**Experimentally determined expiratory time constants for the right chamber plotted against the compliance of the lung chambers.** Both the right and left lung obstructions were held constant at k_L_ = k_R_ = 3.3. The test lung was ventilated with air or He/O_2_ with either a tidal volume of 500 ml at 12 breaths/min, producing minute ventilation of 6 l/min, or a tidal volume of 1000 ml at 20 breaths/min, producing minute ventilation of 20 l/min.

The relative effects of breathing pattern and gas density on expiratory time constants are further explored in Figure 4, in which the time constant of the right chamber is plotted against varying chamber compliance. As both the loss coefficient and the compliance of the two chambers were equal during these measurements, no heterogeneity in ventilation between the two chambers was observed, and the reported right chamber time constants are also representative of the left chamber and whole lung time constants. Referring again to Figure 4, for a given breathing pattern and gas, the time constant increased linearly, or close to linearly, with compliance. Furthermore, despite the fact that these measurements were made with relatively low resistance (one Rp5, representative of healthy airways [11]), time constants were still strongly affected by the breathing pattern and gas density. This is in part a reflection of the added flow- and density-dependent resistance imposed by the breathing circuit, as will be discussed in the following section. However, even when resistance is small, a further reduction caused by decreasing flow rates or decreasing gas density can have a strong influence on the time constant because the time constant arises from the multiplicative influences of resistance and compliance (as in Equation 4 for the idealized single compartment linear model). That is to say, time constants that become large due to loss of pulmonary elasticity that increases compliance (e.g. caused by emphysema) may be reduced by decreasing airway resistance, thereby facilitating lung emptying during exhalation. Given that the resistance of normal, healthy airways is density-dependant [11,19], breathing He/O_2_ may, rather counter-intuitively, improve expiratory flows in cases where elasticity is reduced, but airway resistance is essentially normal. It is also worth noting that the compliances of the left and right chambers were kept equal to one another throughout the present experiments, whereas variation in compliance between lung regions is not uncommon in obstructed patients. While the arguments made above are expected to hold on the regional or compartmental level, heterogeneity in compliance has not been directly studied here.

**Figure 5:**
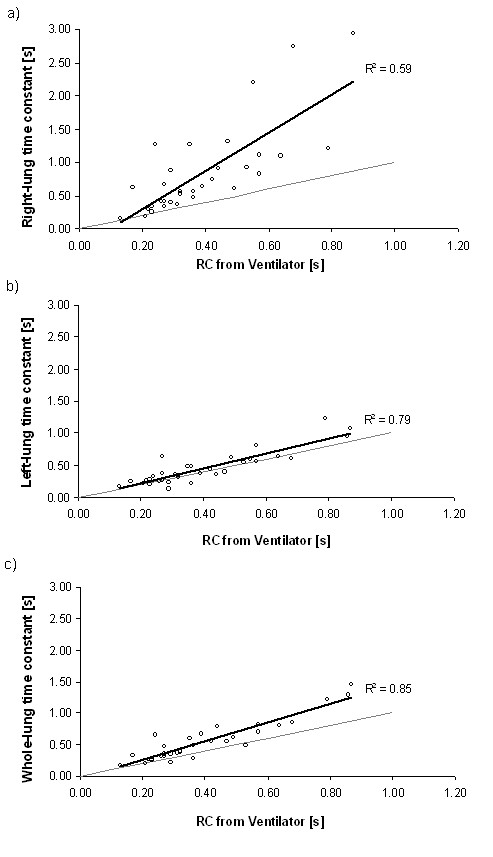
**Experimentally measured expiratory time constants for the (a) right, (b) left, and (c) whole lung, plotted against the expiratory time constant (RC) reported by the ventilator.** The gray lines are identity line, whereas the heavy black lines indicate least-squares linear fits to the data, with corresponding R^2^ values.

Finally, the measured right, left, and whole lung time constants across the full range of experimental parameters studied are plotted in Figure 5 against the expiratory time constant (RC) reported by the ventilator, which is calculated by the ventilator used in the present study as the ratio between the expiratory tidal volume and the expiratory flow rate at 75% of the tidal volume [20]. While the time constant reported by the ventilator is in reasonable agreement with those of the left chamber and the whole lung, it is poorly correlated with the time constant of the right (obstructed) chamber. This result is anticipated given that the ventilator is designed to monitor the total flow into and out of the lung. As the right chamber became more and more obstructed, it received a smaller and smaller fraction of the total tidal volume, and therefore its behavior had increasingly little influence on flows measured at the central airway. Accordingly, changes to the time constant, and associated end-expiratory gas trapping, of poorly ventilated lung compartments may go unnoticed when monitoring flows at the ventilator level. This is similar to the findings of Kurahashi et al. [14], who concluded that the intrinsic positive end-expiratory pressure (PEEPi) of an individual lung compartment can be much greater than that measured at the central airway. In such circumstances, potential effects of He/O_2_ on ventilation heterogeneity are difficult to assess in the clinic. While efforts have been made using the ratio of dynamic to static intrinsic PEEP as an indicator of heterogeneity, this ratio is influenced both by regional variation in time constants and by viscoelastic relaxation of tissue, such that the relative contribution of either factor is not clear [15].

### Resistance imposed by the external breathing circuit

**Figure 6:**
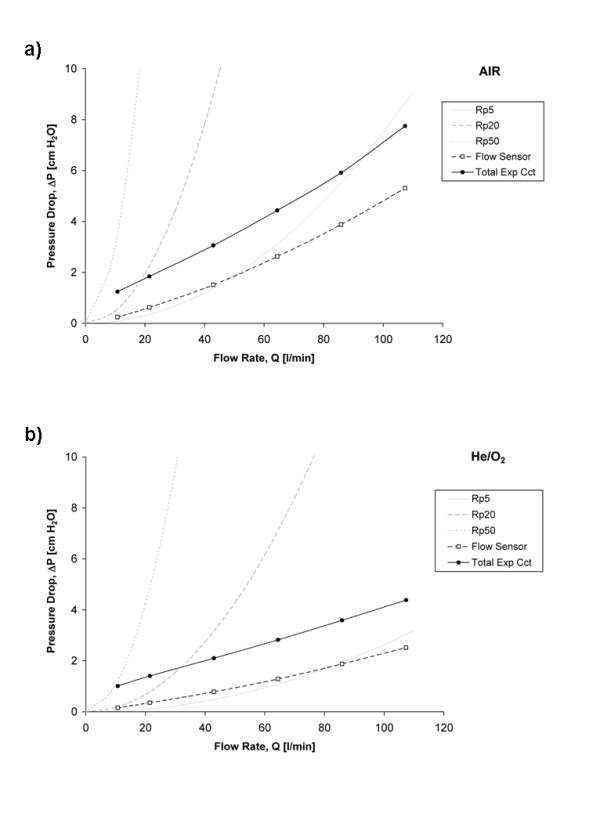
**The pressure drop across the flow sensor, and across the total expiratory circuit (including the flow sensor), plotted against the flow rate of a) air and b) He/O**_**2**_**78/22.** For comparison, the pressure-flow relationships for Rp5, Rp20, and Rp50 resistors are also shown.

**Figure 7:**
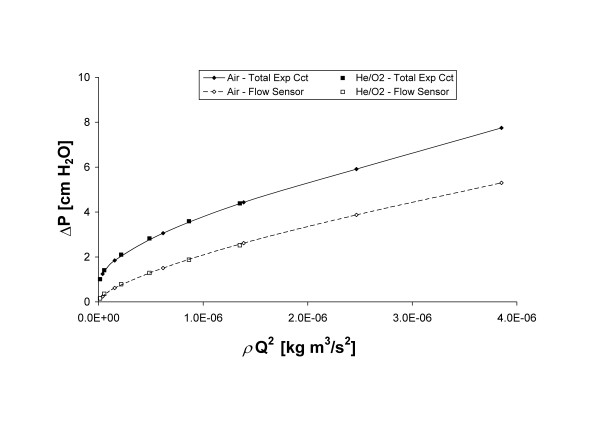
**The pressure drop across the flow sensor, and across the total expiratory circuit (including the flow sensor), plotted against the product of gas density and the square of flow rate for air and He/O**_**2**_.

Figures 6a and 6b show the pressure drop measured across the flow sensor, and across the total expiratory circuit (including the flow sensor), as a function of flow rate of air or He/O_2_, respectively. For comparison, the pressure-flow relationships for the Rp5, Rp20, and Rp50 resistors are also indicated for both gases. The expiratory circuit consisted of the ventilator flow sensor, y-piece, standard 22 mm tubing, and an expiratory valve. Pressure losses across this circuit were dominated by the flow sensor and the expiratory valve. For both these components, the gas passageway grows larger with increasing flow rate (though for the expiratory valve PEEP applied by the ventilator can have an opposing effect), so that the increase in pressure drop with flow is less pronounced than for the rigid parabolic resistors. Nevertheless, as indicated in Figure 7, the pressure losses across both the flow sensor and the expiratory circuit taken as a whole remain dominated by inertial effects, and therefore vary with gas density and with the square of the flow rate. Such a result indicates that the resistance imposed by the breathing circuit at a given expiratory flow rate will be reduced with He/O_2_ compared to air. This reduction would be even more significant with the inclusion of an endotracheal tube for invasive ventilation, as the endotracheal tube would impose an additional flow- and density-dependent resistance [21-23].

### Comparison with analytical model

**Figure 8:**
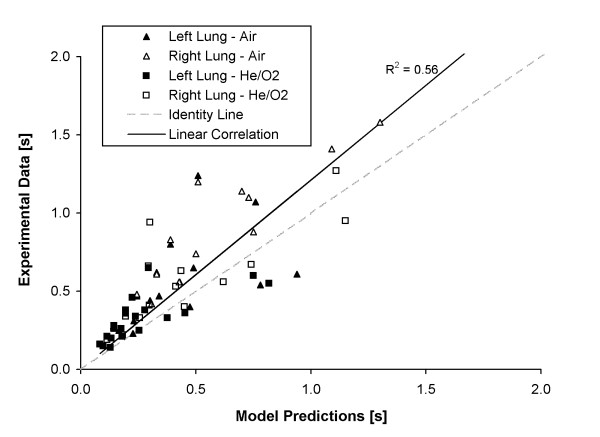
**Comparison between expiratory time constants predicted by the analytical model and those determined experimentally with the two compartment test lung.** The data range is limited to 0–2 s in order to best display the majority of the data.

Figure 8 compares regional time constants predicted analytically for the left and right chambers with those measured experimentally, in both cases following the solution described in Equations (7) and (8). As seen, a reasonable correlation was found between the predicted and measured values. This is an encouraging result, as future versions of the model could include features such as additional lung compartments (e.g., 5 lobes), mixed viscous-inertial airway resistance, and volume-dependant lung compliance that are pertinent to lung mechanics but are more difficult to implement using a mechanical test system. For example, we have previously reported on the extension of the engineering pressure loss model to a multi-generation airway tree by incorporating generation-specific loss coefficients derived from computational fluid dynamics simulation [11]. Further, it is important to note that the present experiments were conducted with dry, room temperature, normoxic gases, whereas gases administered in the clinic are regularly heated and humidified, and often delivered at higher oxygen concentration. While temperature and humidity will have only small influence on gas properties, increasing the oxygen concentration in a He/O_2_ mixture will of course strongly influence its properties [24]. In contrast to repeated experiments on the bench, mathematical modeling offers the potential to rapidly estimate the influence of gas properties on ventilation across a large range of mixture concentrations.

**Figure 9:**
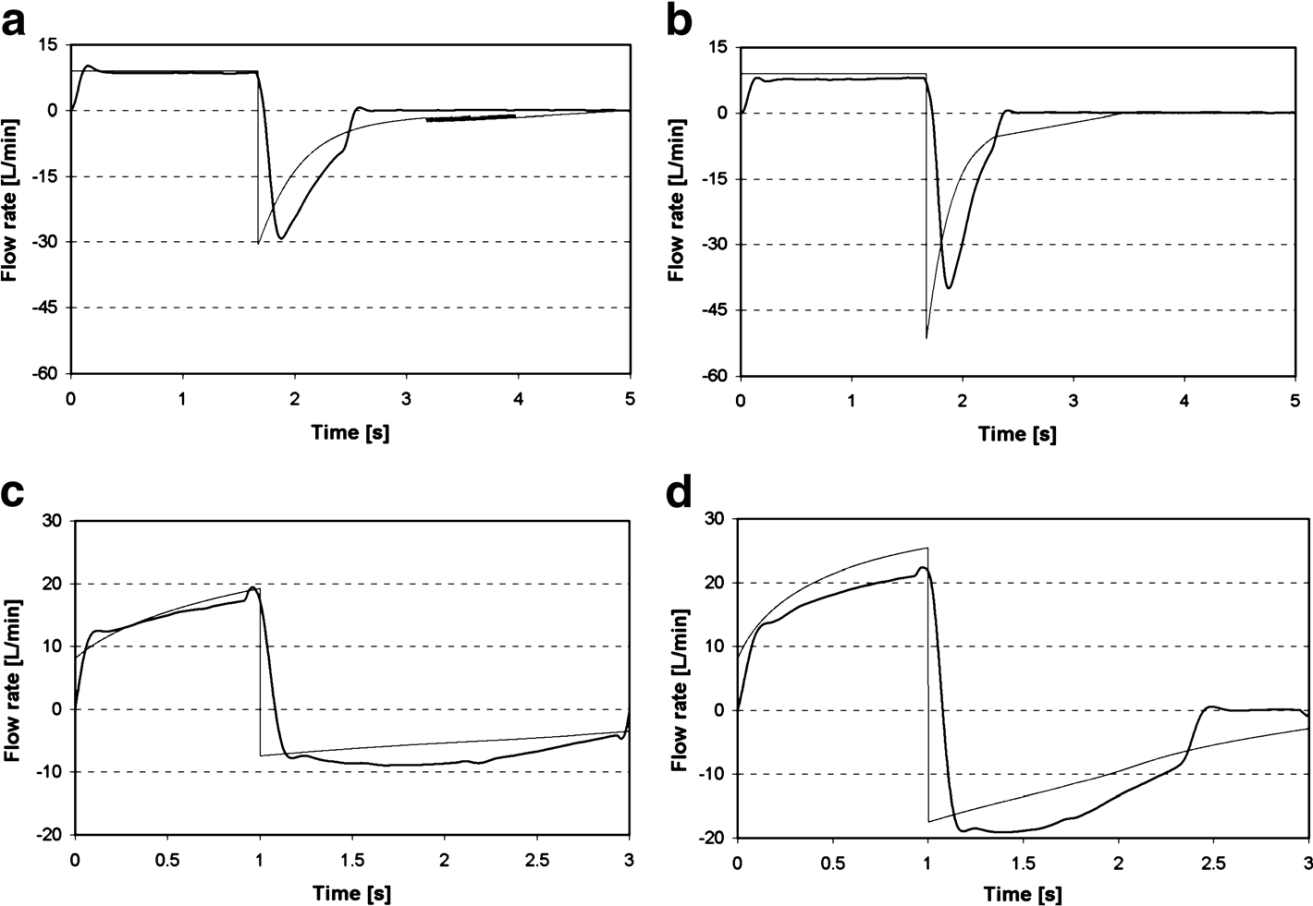
**Flow versus time data measured experimentally (black lines), and predicted analytically (gray lines) for the right lung compartment under several test conditions: a) air and b) He/O**_**2**_** with C**_**R**_** = 0.05 l/cm H**_**2**_**O, k**_**R**_** = 3.3, and Ve = 6 l/min; and c) air and d) He/O**_**2**_**with C**_**R**_** = 0.05 l/cm H**_**2**_**O, k**_**R**_** = 132.9, and Ve = 20 l/min.**

Differences between predicted and measured time constants observed in Figure 8 may be attributed in part to the omission of inertia and friction of the test lung components (e.g. bellows, chamber top plates, hinges) in the model. This can be observed in the curves displayed in Figure 9, where, for example, cycling between inspiratory and expiratory phases is instantaneous for the model, but comparatively delayed for the experiments. Also, for many cases with longer experimental time constants, expiratory flow behavior was clearly not well described by a single exponential function. The case displayed in Figure 9d serves as an example. The modeled and experimental time constants were 0.74 and 1.27 s, respectively. In this case, the experimental curve in particular differs considerably from a single exponential function, to the extent that one might question the applicability of describing such an irregular expiratory curve using a single time constant, at least when determined following the methodology employed herein. Here, it is best to consider the calculated time constant as an index related to expiratory flow from the lung compartment rather than as a mathematical parameter that precisely describes the flow behavior.

## Conclusions

In summary, where inertial phenomena contribute to airway resistance, He/O_2_ reduces expiratory time constants compared to air. Regional variation in resistance leads to regional variation in expiratory flow, and the behavior of obstructed lung compartments has only minor influence on the time constant derived from flow data collected by the ventilator. Accordingly, such measurements may not permit a full appreciation of the effects of He/O_2_ on regional lung ventilation. Finally, results of experiments performed using a mechanical test lung were reproduced by a mathematical model that can be readily extended to include further physiological and pathophysiological phenomena pertinent to the assessment of He/O_2_ therapy for specific diseases and phenotypes.

## Competing interests

All authors are present or past employees of Air Liquide, which has conducted and is presently conducting clinical trials to evaluate helium/oxygen therapies.

## Authors’ contributions

AM conceived of the experimental study, participated in its design, carried out analysis of experimental and analytical data, and drafted the manuscript. IK participated in the design of the study, conceived of and participated in the development of the analytical model, carried out analysis of experimental and analytical data, and edited the manuscript. KT participated in the experimental study design and conducted the experiments. LG participated in the development of the analytical model, and carried out the calculations. GC participated in the design of the study. JT participated in the design of the study and edited the manuscript.
